# Hijacking the bone niche: mechanistic insights into bone metastasis in breast cancer

**DOI:** 10.1038/s41413-026-00547-z

**Published:** 2026-06-10

**Authors:** Jiadi Wu, Min Deng, Feng Ye, Wei Deng, Zichao Wu, Xinhao Zheng, Cailu Song, Yutian Zou, Hailin Tang

**Affiliations:** 1 State Key Laboratory of Oncology in South China, Guangdong Provincial Clinical Research Center for Cancer, Sun Yat-sen University Cancer Center, Guangzhou, China; 2 Guangzhou Institute of Cancer Research, Affiliated Cancer Hospital of Guangzhou Medical University, Guangzhou, China

**Keywords:** Cancer, Bone cancer

## Abstract

Breast cancer is one of the most common malignant tumors worldwide, with metastasis being the leading cause of mortality among patients. Bone is the most frequent site of metastasis in breast cancer, accounting for approximately 70% of metastatic cases. Before bone metastasis, primary breast cancer cells secrete circulating factors (e.g., exosomal RNAs, metabolites, and cytokines) to precondition the bone microenvironment and establish a supportive pre-metastatic niche (PMN). After dissemination, tumor cells further hijack the bone niche by releasing receptor activator of nuclear factor-κB ligand (RANKL), parathyroid hormone-related protein (PTHrP), and transforming growth factor-β (TGF-β), thereby disrupting bone homeostasis through osteoclast activation and osteoblast dysregulation. Bone matrix degradation subsequently releases latent growth factors that in turn fuel tumor cell proliferation, thereby establishing a self-reinforcing vicious cycle. Meanwhile, breast cancer cells actively induce local immunosuppression and promote angiogenesis, remodeling a pro-tumor bone niche conducive to metastatic outgrowth. This review highlights the immunosuppressive roles of immune cells and key molecular mediators in the vicious cycle, systematically analyzes intercellular crosstalk within both the bone PMN and the hijacked niche, and summarizes emerging therapeutic strategies (including novel targeted inhibitors, immune-based combinations, epigenetic modulation, and nanomedicines) beyond conventional treatments. These insights provide a theoretical framework and identify promising therapeutic targets for future treatment strategies against breast cancer bone metastasis.

## Introduction

Breast cancer is the most common malignant tumor in women and one of the most prevalent malignant tumors in the world.^1^ Clinically, breast cancer is classified into four main subtypes: luminal A, luminal B, HER2-enriched, and basal-like (also known as triple-negative) subtypes. Through detection of steroid hormone receptor expression, luminal breast cancer is positive for estrogen receptor (ER) or progesterone receptor (PR), or positive for both ER and PR, and negative for human epidermal growth factor receptor 2 (HER2). The HER2-enriched breast cancer is positive for HER2 expression. The basal-like breast cancer, which is commonly known as triple-negative breast cancer (TNBC), is negative for ER, PR, and HER2 expression.^1^

Distant metastasis is the main cause of cancer-related death in breast cancer patients. Metastatic breast cancer has a poor prognosis, with an overall 5-year survival rate of approximately 32%.^2^ Breast cancer exhibits a distinct metastatic pattern, often showing metastases to the bone, liver, lung, and brain. Different breast cancer subtypes show different preferences for metastatic sites. For instance, luminal breast tumors usually metastasize to the bone, compared with the basal-like subtype, the frequency of bone metastasis in luminal breast cancer is increased by 2.5 times.^3,4^ TNBC usually metastasize to the lung, liver, and brain, and HER2-positive breast cancer shows a propensity for brain and lung and liver metastases.^5^ Luminal breast cancer exhibits a strong predilection for bone metastasis, driven by multiple coordinated mechanisms. The glycoprotein SCUBE2 activates Hedgehog signaling pathway in mesenchymal stem cells (MSCs) within the bone tissue and induces their differentiation into osteoblasts. These newly formed osteoblasts secrete large amounts of collagen, which then inhibits natural killer (NK) cells’ activity through the COL1/LAIR1/SHP1 axis, suppressing immune surveillance.^6^ In addition, the transcription factor FOXF2/RELA/RelA/p65 formed positive feedback, strengthening the ability of luminal breast cancer cells to colonize the bone.^7^ NAT1 is highly expressed in luminal breast cancer, also significantly activates NF-κB, which in turn upregulates the expression of the downstream factor IL-1β, this thereby enhances bone metastasis.^8^

Breast cancer bone metastasis (BCBM) is a major clinical challenge severely affecting patient quality of life and survival. While previous reviews have focused on BCBM, they lack a comprehensive integration of key mechanisms and the latest advances. This review fills these gaps with distinct novelty, integrating BCBM sequential pathways and stage-specific regulatory molecules/genes, incorporating the bone PMN and its role in metastatic initiation, highlighting bone as an immune-privileged site, clarifying the immunosuppression-bone destruction vicious cycle and related immunotherapies, systematically organizing the BCBM-associated non-coding RNA networks and their regulatory effects on tumor-bone-immune cell interactions, and summarizing novel therapeutic strategies (novel signaling pathway inhibitors, immune checkpoint combinations, epigenetic therapies, and targeted nanomedicines). Collectively, this review integrates fragmented knowledge, supplements understudied areas, and provides valuable insights for basic research and clinical translation in BCBM.

## Bone microenvironment

The bone microenvironment is extremely complex, encompassing a variety of hematopoietic cells, stromal cells, osteocytes, osteoblasts, osteoclasts, adipocytes, endothelial cells, mesenchymal cells, and immune cells.^9^ In addition, the bone microenvironment also contains fibroblasts, CXCL12-abundant reticular cells (CAR cells), pericytes, and nerve cells.^10^ Bone remodeling is influenced by the basic multicellular unit, which contains osteoblasts, osteoclasts, bone lining cells, and osteocytes. Osteoblast progenitor cells are recruited from MSCs and differentiate into osteoblasts. Osteoblasts produce osteoid, which matures and mineralizes to form bone tissue.^11^ After bone deposition is completed, some osteoblasts will transform into osteocytes and be embedded in the bone matrix, some may revert to flat cells covering the bone surface, which are called bone lining cells, and some will undergo apoptosis.^12^ Osteoclasts originate from hematopoietic stem cells, wherein monocyte-macrophage lineage cells are stimulated to form osteoclast progenitor cells. These progenitor cells fuse to form multinucleated but non-functional pre-osteoclasts. Under further stimulation, large multinucleated osteoclasts with the ability to resorb bone are produced.^13^ During the physiological bone remodeling process, osteoblasts are responsible for bone formation, while osteoclasts mediate bone resorption. These two cell types are tightly coordinated to maintain the relative stability of bone mass.^14^ When the connection between osteoblasts and osteoclasts is disrupted, it will lead to an imbalance in bone formation and bone resorption, which in turn triggers diseases such as osteoporosis (excessive bone loss) and osteopetrosis (excessive bone formation).^15^

## Breast cancer bone metastasis

Breast cancer cells have a special bone tropism.^16^ Tumor cells are generally prone to metastasize in trabecular-rich bones, including the vertebrae, ribs, and long bones.^14^ In the mouse model of breast cancer metastasis, the tibia is a common site for the formation of secondary tumors. Within the tibia, metastatic tumor cells preferentially localize to the metaphysis rather than the diaphysis.^17^ Several factors contribute to this pattern. Firstly, the diaphysis of long bones is more hypoxic and rarely a site of metastasis, whereas the metaphysis has higher oxygen tension and is usually the site of tumor metastatic lesions.^18^ Secondly, the metaphysis has higher vascular density, which provides essential growth factors and nutrients that support tumor cell proliferation.^14^ Thirdly, compared with other less prone-to-metastasis parts of the bone, the metaphyseal bone tissue has a lower degree of mineral maturity. This difference in mineral properties may be functionally related to the establishment of tumor colonization.^19^

**Fig. 1:**
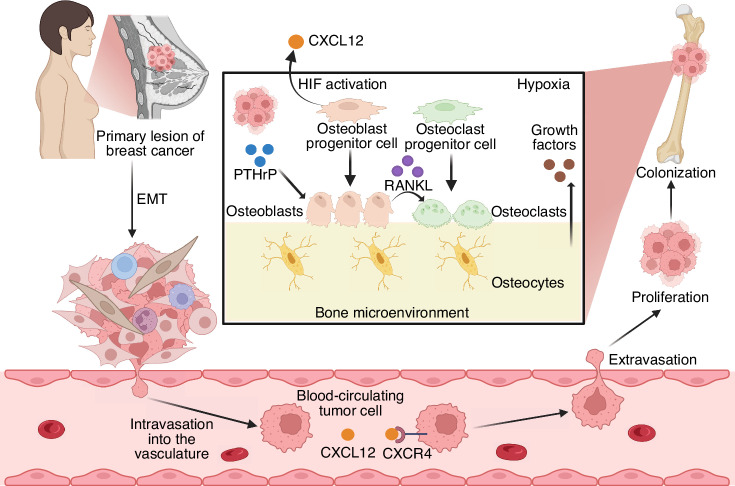
Breast cancer cells metastasize to the bone through the bloodstream. Breast cancer cells acquire the ability to invade through EMT, which enables them to penetrate the basement membrane and enter the circulatory system, thereby spreading to distant organs and forming metastatic lesion. Hypoxia activate HIF in bone progenitor cells and regulate the expression of CXCL12, thereby causing an increase in CXCL12 levels in the circulation. By binding to CXCR4 of breast cancer cells, it further promotes the metastasis of tumor cells to the bone

Due to its abundant blood supply and unique growth microenvironment, the bone is the third most common metastatic site for a variety of solid tumors such as breast cancer, lung cancer, prostate cancer, and colorectal cancer.^20^ Approximately 70% of patients with metastatic breast cancer have bone metastases.^21^ During the process of metastatic dissemination, primary breast cancer cells need to go through a series of complex processes. After acquiring migratory ability through epithelial-mesenchymal transition (EMT), these cancer cells invade the surrounding tissues. Subsequently, they break through the blood vessel wall barrier, infiltrate into the microvessels, and undergo intravasation. Finally, they colonize and form metastatic lesions in the bone tissue (Fig. 1).^22^ Once the cancer spreads to the bones, it is difficult to cure and is accompanied by multiple complications.^14^

The majority of bone metastases from breast cancer are classified as bone-destructive osteolytic lesions, which are associated with a variety of bone complications, such as bone pain, fractures, hypercalcemia, paralysis, and other skeletal-related events (SREs), all of which severely affect the quality of life of patients.^5,23^

## The pre-metastatic niche

**Fig. 2:**
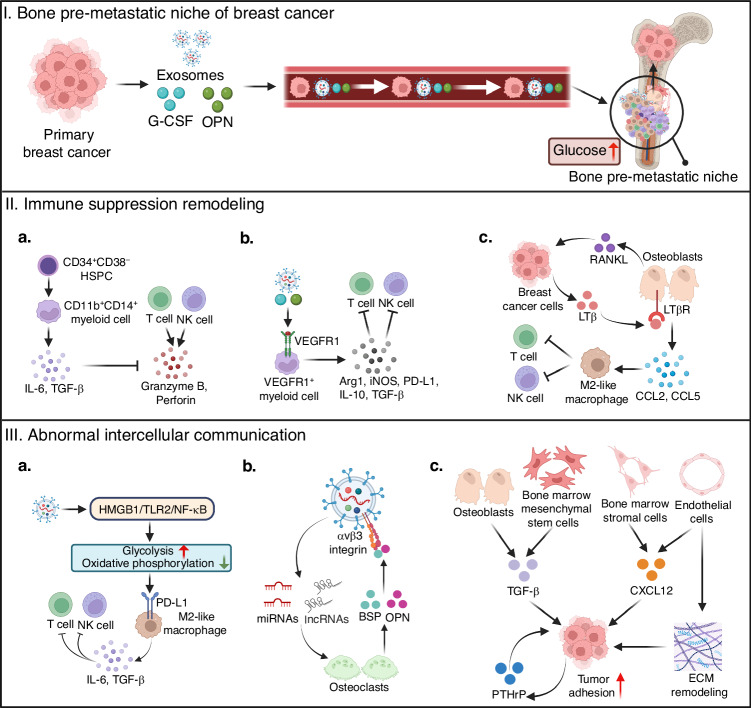
The pre-metastatic niche (PMN) of breast cancer bone metastasis. Before the initiation of the vicious cycle of bone metastasis by breast cancer cells, a specific pre-metastatic niche (PMN) has already formed in the bone tissue. This microenvironment is characterized by metabolic reprogramming, immune suppression, and disrupted intercellular communication. It lays a crucial foundation for the subsequent establishment of a vicious cycle involving tumor cell colonization, bone destruction, and tumor proliferation by remodeling the bone matrix structure, enriching immunosuppressive cells, and activating pro-metastatic signaling pathways. **I.** Primary breast cancer-derived exosomes, G-CSF, and OPN travel through the circulation to remodel the bone microenvironment, establishing a glucose-enriched pre-metastatic niche that supports subsequent tumor cell colonization. **II.** **a** TNBC drives HSPC differentiation into immunosuppressive myeloid cells via IL-6 and TGF-β, remodeling the bone marrow microenvironment to establish the PMN. **b** Tumor-derived G-CSF, OPN, and exosomes recruit VEGFR1⁺ myeloid cells to form an immunosuppressive bone pre-metastatic niche. **c** Tumor cell-derived LTβ, induced by osteoblastic RANKL, promotes chemokine secretion, macrophage recruitment, and tumor cell colonization in the bone PMN. **III.** **a** Tumor-derived exosomes activate the HMGB1/TLR2/NF-κB pathway to induce metabolic reprogramming and PD-L1 expression in macrophages, forming an immunosuppressive bone pre-metastatic niche. **b** Exosomal αvβ3 integrin mediates bone-specific colonization via OPN/BSP binding, while exosomal miRNAs and lncRNAs promote osteoclast activation and inhibit osteoblast function to drive bone matrix degradation. **c** Osteoblasts and MSCs-derived TGF-β, and stromal cells and endothelial cells-derived CXCL12, along with ECM remodeling, enhance tumor cell adhesion and the formation of the bone pre-metastatic niche

The pre-metastatic niche (PMN) of BCBM is a “fertile ground” for the primary breast cancer cells to pre-remodel the bone marrow microenvironment through multiple mechanisms. Its core features include metabolic reprogramming, immune suppression remodeling, and abnormal intercellular communication (Fig. 2). In terms of metabolism, breast cancer creates conditions for tumor colonization by inducing the formation of a high-glucose PMN in bone tissue. Based on this feature, the GPNP@M nano-platform was constructed, which achieves homologous targeting enrichment through tumor cell membranes. The glucose oxidase (GOx) carried can deprive tumor cells of their glucose supply, and in combination with platinum nanoparticles (PtNP), it can achieve precise targeted therapy and block bone metastasis.^24^

In terms of immune suppression remodeling, TNBC can regulate the bone marrow hematopoietic microenvironment, transforming PMN from a normal hematopoietic microenvironment into an immune-suppressive microenvironment. TNBC promotes the differentiation of CD34⁺CD38⁻ hematopoietic stem and progenitor cells (HSPCs) into CD11b⁺CD14⁺ myeloid cells, which inhibit T and NK cell functions by secreting IL-6 and TGF-β, while remodeling the ECM and removing colonization obstacles.^25^ Osteoblasts, endothelial cells, and MSCs work together to regulate by secreting growth factors, modifying the ECM, and inducing myeloid differentiation, respectively, to enhance immune suppression.^25^ Targeted inhibition of the key signals that HSPCs use to differentiate into myeloid cells can prevent the formation of the PMN. Lymphotoxin-β (LTβ) is a key molecule regulating the PMN. Osteoblasts secrete RANKL, which induces tumor cells to express LTβ. LTβ binds to the LTβR on the surface of osteoblasts, promoting tumor colonization, secreting chemokines CCL2/CCL5, recruiting M2-like macrophages, enhancing osteoblast differentiation, and activating dormant tumor cells.^26^ The G-CSF, osteopontin (OPN), and exosomes secreted by tumors travel through the bloodstream to reach the bone marrow and recruit VEGFR1⁺ myeloid cells, forming an immunosuppressive PMN.^27^ The TGFβ, PTHrP, and CXCL12 in the bone microenvironment further enhance the adhesion attraction of the PMN to tumor cells.^17^ Targeting myeloid cells, such as MDSCs, and blocking the chemokine signaling axis can synergistically enhance the effect when combined with osteoclast inhibitors.^28^

In terms of abnormal intercellular communication, the integrins expressed on the surface of tumor exosomes can fuse with target cells in a tissue-specific manner, enabling organ-specific colonization and initiating the formation of the PMN.^29^ In particular, tumor-derived exosomes (TDEs), as a key mediator of intercellular communication, have been proven to be involved in the formation of the PMN. The αvβ3 integrin on the surface of TDEs can bind to OPN and bone sialoprotein (BSP) in the bone matrix, mediating organ-specific colonization and initiating the formation of PMN.^30,31^ TDEs induce metabolic reprogramming in macrophages through the HMGB1/TLR2/NF-κB pathway, resulting in enhanced glycolysis, inhibited oxidative phosphorylation, ultimately causing macrophages to highly express PD-L1, constructing an immunosuppressive PMN.^32^ The miR-494-3p secreted by TDEs can specifically inhibit the expression of leucine-rich repeat G protein-coupled receptor 4 (LGR4) in osteoclast precursors. By regulating the GSK-3β/NF-κB signaling pathway, it enhances the osteoclastogenesis induced by RANKL and accelerates the degradation of bone matrix. At the same time, miR-494-3p directly targets BMPR2 and RUNX2, blocking the BMP-SMAD signaling pathway, inhibiting the differentiation and mineralization ability of osteoblasts, and reducing the synthesis of bone matrix.^33^ Additionally, TDEs also secrete miR-21 to target bone tissue, regulating the programmed cell death protein 4 (PDCD4)/nuclear factor of activated T cells (NFATc1) pathway to activate osteoclasts, inducing bone damage and the formation of osteolytic PMN.^34^ The lncRNA-MIR193BHG secreted by TDEs can be taken up by osteoclasts, promoting osteoclast activation by targeting the miR-489-3p/DNMT3A signaling axis, accelerating bone matrix degradation, and directly constructing an osteolytic PMN.^35^ Therefore, strategies such as targeting the secretion of exosomes and blocking the transmission of specific ncRNAs can intervene in the formation of the PMN.

## Routes and processes of BCBM

BCBM involves a series of sequential and interconnected steps, with multiple cytokines, signaling pathways, and transcription factors participating in and regulating each link. The following will elaborate on the specific processes of BCBM and the role of related molecules in each step.

## Acquisition of metastatic potential by primary breast cancer cells

The first step of BCBM is that primary tumor cells gain invasive and migratory abilities through EMT.^22^ During this process, multiple molecules synergistically regulate the transformation of cancer cells from epithelial to mesenchymal phenotypes. TGF-β is a key inducer of EMT. It can activate the downstream Smad signaling pathway to up-regulate the expression of EMT-related transcription factors (such as Snail, Twist), reduce the expression of epithelial markers (such as E-cadherin), and increase the expression of mesenchymal markers (such as N-cadherin, Vimentin), thereby enhancing the migratory and invasive capabilities of cancer cells.^36^ In addition, the Wnt signaling pathway also participates in the regulation of EMT. Abnormal activation of Wnt/β-catenin signaling can promote the nuclear translocation of β-catenin, combine with TCF/LEF transcription factors to induce the expression of EMT-related genes, and enhance the metastatic potential of cancer cells.^37^ Lysyl oxidase (LOX) can remodel the extracellular matrix (ECM) of the primary tumor, reduce the adhesion between cancer cells, and promote the detachment of cancer cells from the primary tumor mass, providing favorable conditions for subsequent invasion.^38,39^ Matrix metalloproteinases (MMPs), especially MMP-2 and MMP-9, can degrade the basement membrane and ECM components, further enhancing the invasive ability of cancer cells during EMT.^40^

## Invasion of primary tumor cells into surrounding tissues and intravasation into blood vessels

After acquiring mesenchymal characteristics, breast cancer cells begin to invade the surrounding interstitial tissues and eventually break through the vascular wall to enter the circulatory system (intravasation). This process relies on the joint action of matrix-degrading enzymes and cell adhesion molecules. MMPs and cathepsin K play a core role in ECM degradation. MMPs can degrade collagen, fibronectin, and other matrix components, while cathepsin K, as a cysteine protease, has strong collagenolytic activity, which can synergize with MMPs to break down the physical barrier of the surrounding tissues.^12,41^ Platelet-derived growth factor (PDGF) secreted by breast cancer cells can recruit stromal cells (such as fibroblasts) in the surrounding tissues, and the stromal cells secrete more growth factors and proteases in turn, forming a positive feedback loop to promote cancer cell invasion.^42–44^ The Src signaling pathway is involved in regulating the cytoskeletal rearrangement of cancer cells. Activated Src can phosphorylate downstream molecules, promote the formation of pseudopodia and adhesion plaques of cancer cells, and enhance the ability of cancer cells to move and invade the vascular wall.^45,46^ In addition, C-X-C chemokine receptor type 4 (CXCR4) is highly expressed in breast cancer cells,^47^ and bone-derived C-X-C motif chemokine ligand 12 (CXCL12) preferentially recruits breast cancer cells. The interaction between CXCR4 and its ligand CXCL12 can guide cancer cells to move towards blood vessels and promote their adhesion to vascular endothelial cells, laying the foundation for intravasation.

## Circulating tumor cells surviving in the circulatory system and homing to bone tissues

After entering the circulatory system, most circulating tumor cells (CTCs) are cleared by the immune system or die due to shear stress, and only a small number of CTCs can survive and home to bone tissues.^48^ The survival of CTCs is related to the activation of multiple signaling pathways. Insulin-like growth factor (IGF) can bind to the IGF receptor on CTCs, activate the PI3K/Akt signaling pathway, inhibit apoptosis, and promote the survival of CTCs.^49^ The homing of CTCs to bone is a tissue-specific process mediated by chemokine gradients and adhesion molecule interactions. The bone marrow microenvironment is in a hypoxic state, and hypoxia-inducible factor (HIF) in osteoblast progenitors is activated to up-regulate the expression of CXCL12. The high expression of CXCR4 on breast cancer cells enables CTCs to be specifically recruited to the bone marrow under the chemotaxis of CXCL12.^50^ In addition, bone marrow stromal cells secrete granulocyte colony-stimulating factor (G-CSF) and macrophage colony-stimulating factor (M-CSF), which can up-regulate the expression of vascular cell adhesion molecule 1 (VCAM-1) on vascular endothelial cells in the bone marrow, and strengthen the adhesion between CTCs and vascular endothelial cells through the interaction with α4β1 integrins on CTCs, thereby promoting the extravasation of CTCs into the bone marrow microenvironment.^51,52^

## Colonization and proliferation of tumor cells in bone tissues

**Fig. 3:**
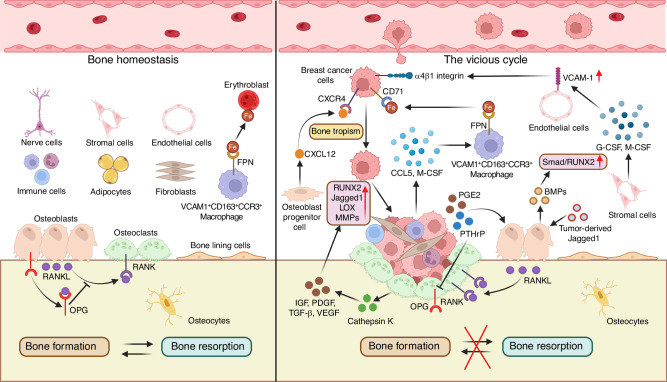
Bone microenvironment and vicious cycle. Under physiological conditions, bone remodeling maintains homeostasis within the bone microenvironment. When VCAM-1 is upregulated on vascular endothelial cells, it interacts with integrins on the surface of breast cancer cells, promoting cancer cell extravasation from the bloodstream and invasion into the bone microenvironment. This disrupts homeostatic balance: bone resorption is increased, bone formation is suppressed, and large amounts of growth factors are released, further stimulating tumor cell proliferation. Tumor-derived PTHrP induces osteoblasts to secrete RANKL, which binds to RANK on osteoclasts, forming a vicious cycle and exacerbating bone destruction. Furthermore, tumor cells secrete factors such as CCL5 and M-CSF to recruit VCAM1⁺CD163⁺CCR3⁺ macrophages. These macrophages switch from their normal physiological function of supporting erythropoiesis to supplying iron to tumor cells. Macrophages release iron via the iron transporter FPN, and tumor cells take up iron through CD71 to support their proliferation

After extravasation into the bone marrow, breast cancer cells colonize and proliferate to form metastatic lesions, which are closely related to the interaction between tumor cells and the bone microenvironment (osteoblasts, osteoclasts, stromal cells). The RANKL/RANK/OPG system is the core regulatory axis of bone metabolism and plays a key role in the formation of osteolytic metastases. Breast cancer cells secrete PTHrP, which can stimulate osteoblasts to secrete RANKL and inhibit the secretion of osteoprotegerin (OPG).^53^ RANKL binds to RANK on the surface of osteoclast precursors, promotes the differentiation and activation of osteoclasts, and enhances bone resorption.^54,55^ Bone resorption releases a large amount of growth factors stored in the bone matrix, which in turn stimulate the proliferation of breast cancer cells and further promote the secretion of PTHrP by cancer cells, forming a “vicious cycle” of bone destruction and tumor growth.^56^ Tumor cells hijack the VCAM1⁺CD163⁺CCR3⁺ macrophages that originally were responsible for supplying iron to erythroblasts in bone metastasis sites by secreting CCL5 and M-CSF. On one hand, the iron transport protein FPN on macrophages provides iron for the tumor, while tumor cells acquire iron through CD71 to promote proliferation. On the other hand, tumor cells activate the GATA1 transcription factor through HIF-1α, upregulate hemoglobin synthesis to simulate the characteristics of erythroblasts, and enhance survival.^57^ At the same time, bone morphogenetic proteins (BMPs) can bind to receptors on breast cancer cells, activate the Smad signaling pathway, and regulate the proliferation and differentiation of cancer cells.^58^ The transcription factor Runt-related transcription factor 2 (RUNX2) is also involved in this process. It can up-regulate the expression of genes related to bone metastasis in breast cancer cells, and promote the adaptation and proliferation of cancer cells in the bone microenvironment.^59,60^ In addition, breast cancer cells secrete Jagged1, which can activate the Notch signaling pathway in osteoblasts through contact-dependent signaling, inhibit osteoblast differentiation, and further promote bone resorption (Fig. 3).^61^

## The “vicious cycle” of bone metastasis

The early colonization of metastatic tumor cells seems to rely on active osteogenesis.^62^ The proliferation and adhesion of metastatic breast cancer cells increase on more immature hydroxyapatite crystals (the main inorganic component of bone tissue),^63^ these early changes in bone minerals promote the initial seeding and survival of disseminated tumor cells (DTCs). During the process of development from early colonization to eventual large-scale metastasis, osteoclasts are aberrantly activated, and the osteolytic activity is enhanced.^23^ This process is tightly regulated by the crosstalk between tumor-derived factors and bone cell signaling. For instance, tumor-secreted Jagged1 can also act on osteoclast precursors to promote their differentiation,^61^ while the Wnt signaling pathway interacts with Src kinase to amplify osteoclast activation.^64,65^ Activated Wnt signaling enhances Src-mediated phosphorylation of downstream targets, which in turn upregulates osteoclast-specific genes, and Src reciprocally modulates Wnt ligand secretion by tumor cells to sustain this pro-osteolytic signal.^65^ At the same time, primary tumor cells also secrete circulating factors, including RUNX2 and LOX, to promote local osteogenesis, thus changing the composition and structure of the ECM of bone cells.^19^ RUNX2 further drives the expression of Jagged1 and MMPs in tumor and osteoprogenitor cells,^61^ while MMPs degrade the ECM to facilitate the infiltration and colonization of tumor cells.^66^ LOX cooperates with RANKL to mediate osteoclast differentiation, thereby accelerating osteoclast bone resorption and the formation of metastatic osteolytic lesions.^67^ Tumor-derived LOX also induces the massive secretion of the osteoclast-promoting cytokine IL-6, and exacerbates osteoclast-mediated bone resorption.^68^

As tumor cells further proliferate and metastasize, they secrete more cytokines and mediators, which further enhance osteoclastogenesis or impair osteoblast function, perpetuating the cycle. Specifically, in BCBM, tumor-derived PTHrP and prostaglandin E2 (PGE2) upregulate RANKL expression in osteoprogenitor cells while concurrently suppressing OPG production. The reduction of OPG amplifies RANKL activity, thereby promoting osteoclast activation and ultimately leading to increased bone resorption.^69,70^ During the bone resorption process, once osteoclasts are activated, they form resorption lacunae and degrade the bone matrix by releasing acids and proteolytic enzymes.^71^ Osteoclasts secrete cathepsin K to degrade bone matrix collagen and release various growth factors (e.g., TGF-β, IGFs) sequestered in the bone matrix.^12,72^ The released growth factors drive tumor cell proliferation, upregulate the expression of RUNX2, Jagged1, LOX, and MMPs.^14^ This integrated network of signals operates reciprocally; tumor cells drive abnormal bone remodeling via these molecules, and the remodeled bone microenvironment releases factors that further stimulate tumor growth and factor secretion. This cycle repeats itself, constantly exacerbating bone destruction and tumor progression, thus forming a vicious cycle (Fig. 3).

## Vascular niche and osteogenic niche

During bone metastasis, after tumor cells extravasate from the bloodstream into the bone marrow, they preferentially colonize two specialized microenvironmental niches: the vascular niche and the osteogenic niche. The vascular niche consists of bone marrow stromal cells, pericytes, and endothelial cells, while the osteogenic niche, which is centered around osteoblasts and osteoclasts, is essential for the regulation of bone remodeling. Both niches play indispensable roles in facilitating cancer cell colonization and progression within the bone.^5^ Once tumor cells are colonized in the bone, the vascular niche continues to play an important role in tumor progression. On the one hand, it provides a suitable dormant environment for cancer cells. On the other hand, as a channel for transporting nutrients and growth factors, it supplies energy for tumor growth.^73^ In the initial stage of bone metastasis, DTCs interact with components of the niche, activate the pro-survival signaling pathway within the cells,^62^ and immune escape.^74^ Tumor cells will also sense various signals in the microenvironment and decide whether to initiate the lesion growth process or enter a dormant state.^75^ In breast cancer, the formation of metastatic lesions is closely associated with the aberrant activation of osteoclasts. Once osteoclasts are activated, they will not only degrade bone tissue but also release bone-binding molecules, thus forming a feedback loop that stimulates cancer cell proliferation.^61^

## Osteomimicry

To adapt to the bone microenvironment, metastatic breast cancer cells express bone-related genes and acquire osteomimetic traits that support their survival and growth within the bone niche. This phenomenon is known as osteomimicry.^76^ Osteomimetic factors include RUNX2, Cadherin-11 (CDH11), osteonectin, BSP, and OPN, etc. For instance, RUNX2 enhances the bone metastasis ability of breast cancer cells, enabling cancer cells to acquire osteoblast-like characteristics.^60^ Knocking it down inhibits osteoclastogenesis and osteolytic lesions in highly invasive breast cancer.^77^ CDH11 and integrin subunit alpha 5 (ITGA5) carried by extracellular vesicles secreted by tumor cells synergistically establish a PMN, significantly promoting the colonization of breast cancer cells with high RUNX2 expression in bone tissue, thus accelerating the process of bone metastasis.^78^ In breast cancer, upregulating osteonectin expression may be a crucial factor driving cancer cells to preferentially home to bone tissue.^79^ The expression level of BSP in breast cancer cells from patients with bone metastasis is significantly higher than that in patients without bone metastasis, confirming the association between BSP and bone metastasis.^80^ OPN is produced by many breast cancer cells and has a strong correlation with poor prognosis, which is involved in the differentiation and activity of osteoclasts, and the inhibition of mineral deposition in the bone-like matrix.^81^

## The release of growth factors

The bone matrix not only provides stable structural support for resident bone cells but also contains a large number of growth factors (Table 1).^82–89^ During the normal bone remodeling process, these growth factors are released and play an important role in maintaining bone health. However, these growth factors also potentially become “accomplices” that promote the growth of bone metastatic tumor cells.^14^ For example, activated osteoclasts secrete TGF-β, IGF, PDGF, and BMP, which promote the production of PTHrP, providing support for the survival and development of tumor cells.^56^ Osteoblast-derived hepatocyte growth factor (HGF),^90^ IL-6,^91^ and connective tissue growth factor (CTGF)^17^ also promote the generation of osteoclasts and the proliferation of tumor cells. In addition, elevated sympathetic nerve outflow activates β2-adrenergic receptors (β2AR) on osteoblasts, which upregulate vascular endothelial growth factor (VEGF) expression in primary tumors and enhance vascular density, thereby favoring metastatic breast cancer cells to colonize and form metastases in the bone.^92^

**Table 1:** The potential growth factors released by osteoclasts in bone metastasis

Growth Factor	Receptor	Effects	Bone formation	Bone resorption	References
TGF-β	TβR-Ⅰ/Ⅱ	PTHrP ↑, RANKL ↑ , OPG ↓	Decrease	Increase	^82^
IGF	IGF-IR	PTHrP ↑, RANKL ↑ , OPG ↓	Decrease	Increase	^83^
PDGF	PDGFR	PTHrP ↑, RANKL ↑ , OPG ↓	Decrease	Increase	^84^
BMP	BMPR-I/II	RUNX2 ↑ , RANKL ↑ , OPG ↓	Decrease	Increase	^85^
CTGF	Integrin αvβ3	RUNX2 ↑ , RANKL ↑ , OPG ↓	Decrease	Increase	^86^
HGF	c-Met	RANKL ↑ , OPG ↓	Decrease	Increase	^87^
IL-6	IL-6R	RANKL ↑ , OPG ↓	Decrease	Increase	^88^
VEGF	VEGFR	RANKL ↑ , OPG ↓	Decrease	Increase	^89^

## Bone is an “immune-privileged site”

Compared with the peripheral circulation, bone is an immune-privileged site characterized by a low abundance of cytotoxic T and NK cells, alongside a high proportion of regulatory T cells (Tregs) and myeloid-derived suppressor cells (MDSCs). Consequently, the bone microenvironment is generally favorable for tumor cell seeding and immune evasion.^14^ Immune cells in bone play an important role in bone remodeling through OPG, RANKL, and other factors. B and T cells are sources of OPG and RANKL, and affect the ratio of RANKL/OPG during homeostasis, aging, and pathological bone loss.^93^ Notably, simply blocking RANKL triggers a significant remodeling of the tumor immune microenvironment, characterized by a substantial increase in the activation levels of dendritic cells (DCs) and plasmacytoid DCs (pDCs), accompanied by a sharp decrease in the number of Treg cells.^94^ Neoadjuvant chemotherapy activates effector T cells and promotes their infiltration into the tumor microenvironment (TME), thus generating a synergistic effect with RANKL blockade and further enhancing the anticancer efficacy.^94^ Under basal conditions, B cells promote osteoclastogenesis, whereas under pro-inflammatory conditions, they inhibit osteoclasts formation.^95^ Similarly, T cells exert dual effects on bone loss: T cells producing tumor necrosis factor alpha (TNF-α) and IL-17 promote the formation of osteoclasts,^96,97^ whereas Tregs inhibit osteoclasts by secreting cytokines such as IL-10 and TGF-β.^98^ Interferon gamma (IFNγ) produced by T cells also balances the formation of osteoclasts by interfering with the RANK signaling pathway.^99^ The Th2 type immune response promotes osteoblast activity and bone formation through the production of parathyroid hormone (PTH), while the Th1 type immune response inhibits osteoblasts through IFNγ.^100^

The study indicates that approximately 27% to 40% of newly diagnosed patients with localized breast cancer already have breast cancer metastatic cells in their bone marrow. Compared with other metastatic sites with relatively robust immune surveillance, the immune-privileged bone marrow microenvironment provides a survival niche for hematopoietic stem cells, while also protecting disseminated breast cancer cells and maintaining them in a dormant state. The dormant state enables the tumor cells to evade immune clearance and chemotherapy effects, subsequently colonizing and proliferating to form visible bone metastatic lesions.^101^ In contrast to the lung and brain, where breast cancer cell colonization relies heavily on angiogenesis and tissue invasion, breast cancer cells colonize the bone marrow by mimicking bone cells (osteomimicry) and hijacking pathways that regulate HSC homing and proliferation to integrate with the bone marrow vascular niche. Among them, CXCR4/CXCL12 play a key regulatory role in the homing, dormancy, and colonization of breast cancer cells in the immune-privileged area of the bone marrow. Moreover, unlike the liver microenvironment, which is characterized by high levels of pro-inflammatory cytokines (e.g., TNF-α, IL-1β) that restrict tumor cells’ survival, the bone marrow is enriched with immunosuppressive factors (e.g., IL-6, TGF-β) that breast cancer cells exploit to adapt to the niche and accelerate metastatic colonization.^102^

Compared with other metastatic sites where immune cells maintain partial anti-tumor activity, the bone’s immune-privileged properties render local immune cells susceptible to manipulation by breast cancer cells, transforming them into “helpers” that promote colonization. Research has found that breast tumors can induce bone marrow-derived CD19^+^ B cells to express RANKL. These B cells, in collaboration with tumor-activated CD3⁺ T cells, will trigger bone resorption and bone loss, disrupt bone homeostasis, while providing colonization space for breast cancer cells. In addition, unlike DCs in the lung that primarily prime anti-tumor T cell responses, bone marrow DCs assist the activity of RANKL⁺ T cells and differentiate into osteoclast-like cells, which retain antigen-presenting functions and form a positive feedback loop of osteolytic effects, further accelerating breast cancer cell colonization. Compared with T cells in the liver and brain metastatic niches that retain partial cytotoxicity, T cells in the bone microenvironment exhibit profound functional inhibition, and the bone resorption mediated by them will instead promote the proliferation of breast cancer cells and exacerbate bone metastasis colonization.^103,104^ Moreover, unlike cancer cells in the lung that often upregulate MHC-I to evade immune escape, breast cancer cells in the bone microenvironment downregulate MHC-I expression, making it more difficult for T cells to recognize and kill them, thereby extending the window for initial colonization.^105^ Mononuclear myeloid suppressor cells (M-MDSCs) highly express PD-L1, and approximately 70% of T cells in bone metastasis lesions express PD-1. The combination of these two directly inhibits T cells, promotes the formation of osteoclasts, and thereby exacerbates bone metastasis.^106^

**Fig. 4:**
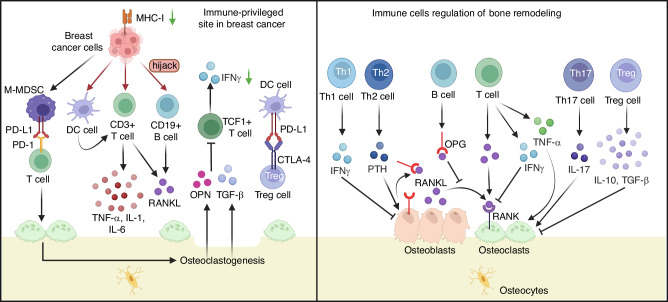
The characteristics of the bone marrow immune-privileged site in breast cancer and immune cells in the bone microenvironment are involved in bone remodeling. In the bone microenvironment, T cells are an important source of RANKL, while B cells are an important source of OPG. Osteoblasts, T cells and B cells jointly participate in the regulation of the ratio of RANKL/OPG, which serve as an important “intersection point” connecting bone metabolism and the immune system. Th1, Th2, Th17 and Treg cells also play significant roles in bone metabolism by secreting various cytokines. Among them, IFNγ has a dual function, not only inhibits osteoclasts to prevent excessive bone destruction, but also inhibits osteoblasts to reduce bone formation. When breast cancer cells metastasis the bone microenvironment, cancer cells downregulate MHC-I to evade immune recognition. They recruit M-MDSC to strongly inhibit T cell function through the PD-1/PD-L1 pathway. Moreover, tumor cells “hijack and reconfigure” immune cells to drive bone resorption, and immune suppression further amplifies the osteoclastogenic effect, continuously accelerating the process of breast cancer bone metastasis

The immune-privileged sites of bones play a significant regulatory role in the efficacy of immunotherapy under tumor bone metastasis. Metastatic cancer cells activate the RANKL/RANK signaling pathway to induce abnormal activation of osteoclasts, which secrete large amounts of OPN into the bloodstream, remotely inhibiting the proliferation and function of CD8⁺ T cells in bone metastatic lesions, especially TCF1⁺ precursor exhausted T cells, inducing the formation of an “immune cold tumor” at the lesion site and reducing the efficacy of immune checkpoint blockade (ICB).^107^ Additionally, compared with the relatively low TGF-β levels in the lung microenvironment, bone metastatic lesions have high concentrations of TGF-β that directly induce CD8⁺ T cells exhaustion and reduce IFN-γ secretion. Concurrently, Tregs inhibit the maturation of DCs through the CTLA-4/PD-L1 pathway, resulting in the inability of ICB to activate effective anti-tumor immunity (Fig. 4).^108^ Above mentioned the study not only provides a new perspective for understanding the impact of bone metastasis on the systemic immune response, but also offers potential therapeutic strategies for improving the ICB treatment effect in patients with bone metastasis.

## Wnt signaling

The Wnt signaling pathway is a highly ordered multi-step signaling cascade system, playing a crucial role in numerous physiological and pathological processes, especially in the process of bone metastasis.^109^ The intracellular activation modes of this pathway are mainly divided into two categories: the β-catenin-dependent pathway (canonical pathway) and the β-catenin-independent pathway (non-canonical pathway). During the activation process of the typical β-catenin-dependent Wnt pathway, the Wnt ligand binds to the low-density lipoprotein receptor-related proteins 5/6 (LRP5/6) and Frizzled (FZD) receptors on the cell membrane surface, triggering a series of downstream reactions: activating the Dishevelled (Dvl) protein, inhibiting the activity of glycogen synthase kinase 3β (GSK3β), and simultaneously affecting the functions of the Axin protein and the adenomatous polyposis coli (APC) protein. This series of changes allows β-catenin, which was originally degraded in the cytoplasm, to accumulate and enter the cell nucleus. In the nucleus, β-catenin binds to members of the nuclear transcription factor T cell factor (TCF) family and the lymphoid enhancer factor (LEF) family to regulate the transcriptional expression of downstream target genes.^110^ The β-catenin-independent Wnt pathway consists of multiple branches, such as the Wnt/Ca²⁺ pathway, the mammalian target of rapamycin (mTOR) pathway, the Ras homolog gene family member A/rho-associated protein kinase (RhoA/ROCK) pathway, and the c-Jun N-terminal kinase (JNK) pathway.^111^ There are multiple mechanisms for the inactivation of the Wnt pathway: On the one hand, Wnt inhibitory factor (WIF) or secreted Frizzled-related proteins (SFRPs) bind to the Wnt ligand, blocking the interaction between the ligand and the receptor. On the other hand, Dickkopf 1 (DKK1) or sclerostin (SOST) bind to the LRP5/6 receptor, hindering signal transmission.^112^ DKK1 as a downstream target gene of the typical Wnt pathway, significantly increases its secretion amount when the Wnt pathway is over-activated, thereby forming a negative feedback regulation on the Wnt pathway to prevent excessive signal transduction.^113^

**Fig. 5:**
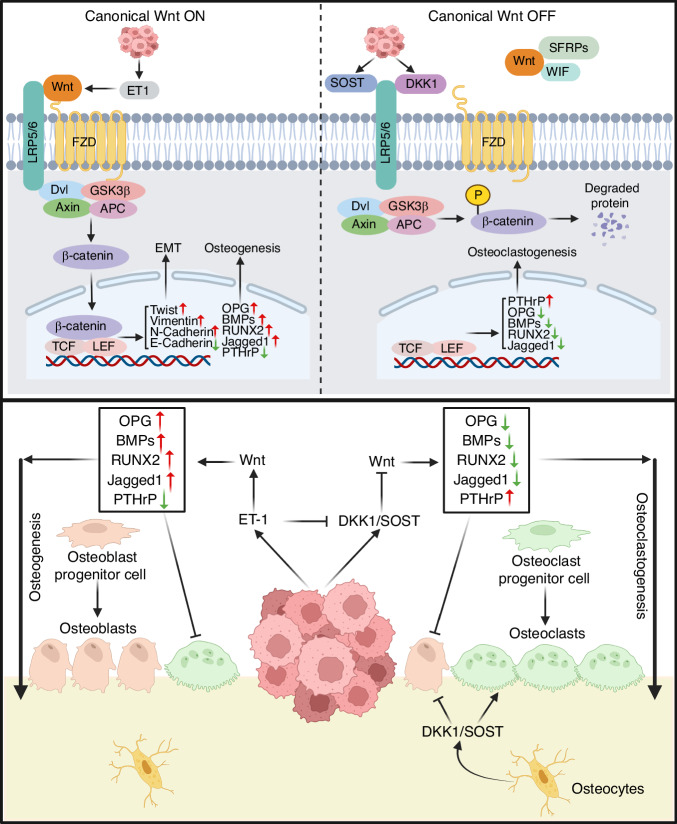
The dual role of the Wnt pathway in tumor cells and the bone microenvironment. In the on state, under the action of ET-1, Wnt ligands bind to LRP5/6 and FZD receptors, activate Dvl, inhibit GSK3β, and affect the functions of Axin and APC, allow β-catenin to accumulate in the nucleus, binds to the TCF/LEF family and regulates the transcription of EMT and osteogenesis related genes. In the off state, WIF/SFRPs bind to Wnt ligands and DKK1/SOST bind to LRP5/6 both inhibit the activation of the Wnt pathway, the activity of the degradation GSK3β complex is retained, β-catenin is degraded, unable transcript the EMT and osteogenesis related genes. Breast cancer cells inhibit the Wnt pathway by secreting DKK/SOST, which suppress osteoblast activity, promote osteoclast differentiation, intensify osteolytic destruction. However, blocking DKK/SOST, led to aberrant osteogenesis

The canonical Wnt signaling pathway plays a central role in the bidirectional communication between cancer cells and the bone microenvironment. Breast cancer cells secrete molecules that can either activate or inhibit the Wnt pathway, disrupting the delicate balance between bone formation and resorption. Simultaneously, Wnt-related factors produced by the bone microenvironment influence cancer cells, further promoting their malignant progression.^114^ Cancer cells secrete SOST, which significantly reduces the activity of osteoblasts by inhibiting the Wnt/β-catenin pathway.^115^ Breast cancer cells secrete DKK1, bind to LRP5/6 and Kremen-1, inhibit the expression of OPG and osteoblast differentiation, promote the generation of osteoclasts, and accelerate the progression of osteolytic bone metastasis.^116^ Meanwhile, under the condition of bone resorption, osteocytes also secrete SOST and DKK1 to inhibit the differentiation and function of osteoblasts.^117^ Blocking SOST activates the canonical Wnt signaling, ultimately exacerbating BCBM.^64^ Breast cancer cells also secrete ET1, which inhibits DKK-1, thereby enhancing the activity of osteoblasts and promoting uncontrolled bone formation (Fig. 5).^118^

## Non-coding RNA

Circular RNAs (circRNAs) play pivotal roles in BCBM by regulating key molecular pathways, interacting with microRNAs (miRNAs), and modulating the TME.^119^ Research has found that circMMP2(6,7) is significantly upregulated in bone-metastatic breast cancer tissues, which form a complex with β-catenin and PRMT5 to activate bone‑remodeling genes (*S100A4*, *LGALS3*) via histone arginine methylation, promoting osteoclastogenesis, osteoblast suppression, and significantly enhancing bone tropism of breast cancer cells.^120^ CircIKBKB is also significantly upregulated in bone-metastatic breast cancer tissues, which promotes IKKβ-mediated IκBα phosphorylation and activates the NF-κB pathway, significantly promoting osteoclastogenesis and bone PMN formation in breast cancer.^121^ CircRBMS3 is elevated in bone-metastatic breast cancer and linked to worse 3-year survival. Knocking it down inhibits cancer cell growth and osteoclast-related gene expression. CircRBMS3 binds miR-654-3p, revealing a novel regulatory axis in bone metastasis.^122^

The interplay between miRNAs is fundamental to their regulatory roles in BCBM, which involves upstream competing endogenous RNAs (ceRNAs) such as long noncoding RNAs (lncRNAs) and circRNAs, downstream target mRNAs, RNA-binding proteins, and crosstalk with other miRNAs.^123^ CircRBMS3 is elevated in bone-metastatic breast cancer, correlates with poor prognosis, and drives bone metastasis via the circRBMS3/miR-654-3p/RANKL ceRNA axis, which enhances bone tropism of breast cancer cells and induces osteoclast activation to remodel the bone PMN.^122,123^ Previous studies have confirmed that lncRNAs can regulate BCBM through the ceRNA model.^124^ For example, lncRNA MIR193BHG directly binds to miR-489-3p within osteoclasts, and the inhibitory effect of miR-489-3p on DNMT3A is relieved, resulting in high expression of DNMT3A in osteoclasts, promoting osteoclast activation, accelerating bone resorption, and ultimately promoting BCBM.^35^ LncRNA SNHG3 directly binds to miR-1273g-3p within bone marrow mesenchymal stem cells (BMSCs), and the inhibitory effect of miR-1273g-3p on BMP3 is relieved, leading to increased expression of BMP3 in BMSCs, further inhibiting osteogenic differentiation of BMSCs, disrupting the “osteogenesis-osteolysis balance”, and ultimately promoting osteolytic BCBM.^125^ LncRNA TRG-AS1 competitively binds to miR-877-5p, allowing miR-877-5p to no longer inhibit WISP2, thereby maintaining the normal level of WISP2, restoring the osteogenic differentiation ability of BMSCs, inhibiting osteoclast activation, and ultimately blocking BCBM.^126^ LncRNA MALAT1 directly binds to the Tead3, preventing it from forming a complex with NFATC1. thereby inhibiting the gene transcription mediated by NFATC1, ultimately blocking the differentiation of osteoclast precursor cells into mature osteoclasts.^127^

MiRNAs play a crucial role in the occurrence, development, metastasis of tumors, and the acquisition of resistance to treatment regimens,^128,129^ especially for the treatment of bone metastases.^130^ MiR-10b as a metastasis-initiating miRNA in breast cancer and drives bone invasion and metastasis via the miR-10b/HOXD10/RhoC axis.^131^ MiR-214-3p is elevated in bone-metastatic breast cancer and directly targets Traf3, an inhibitor of osteoclast differentiation, thereby enhancing the bone resorption process.^132^ In addition, miR-218-5p is also elevated in bone-metastatic breast cancer. As an important oncogenic factor, it promotes bone metastasis of TNBC cells via repressing Wnt signaling activation through DKK1/SFRP1.^133^

MiRNAs not only have the ability to promote bone metastasis, but also possess the function of inhibiting bone metastasis. For example, miR-124 is a pivotal tumor suppressor that inhibits BCBM via direct repression of IL-11, disrupting the IL-11/STAT3 osteoclast axis and breaking the cancer-bone vicious cycle.^134^ MiR-135 and miR-203 prevent the formation of osteolytic lesions in breast cancer by directly targeting RUNX2.^135^ The miR-30s family precisely targets multiple key genes, such as genes related to osteoclastogenesis stimulation (*IL11*, *TWISIT1*), genes related to osteoblastogenesis inhibition (*DKK-1*), genes related to tumor cell osteosomatosis (*RUNX2*, *CDH11*), and genes related to invasiveness (*CTGF*, *ITGA5*, *ITGB3*), thereby inhibiting the process of BCBM.^136^ MiR-34a acts as a tumor suppressor in TNBC by directly silencing c-Src and inhibiting PI3K/Akt and MAPK/ERK oncogenic signaling pathways, thereby blocking TNBC progression to bone metastasis.^137^ MiR-429 inhibits the occurrence of BCBM by directly targeting V-crk sarcoma virus CT10 oncogene homologous protein (CrkL) and MMP-9.^138^ MiR-506 is a pivotal tumor suppressor that ameliorates breast cancer-induced osteolytic bone metastasis by repressing the NFATc1 signaling pathway, disrupting both osteoclast activation and cancer cell bone colonization.^139^ In addition, Overexpressed miR-223-3p effectively reduces the osteoclast formation of breast cancer by inhibiting the production of MDK.^140^ In conclusion, ncRNAs have the potential to become therapeutic targets and prognostic markers, and have broad application prospects in combating and monitoring BCBM and improving the survival status of patients (Table 2).^35,120–122,125–127,132–142^

**Table 2:** The effects of ncRNAs on bone metastasis of breast cancer

ncRNAs	Targets	Effects	References
circMMP2(6,7)	β-catenin/PRMT5	Bone metastasis ↑	^120^
circIKBKB	IKKβ activation ↑, IκBα phosphorylation ↑	Osteoclastogenesis ↑, Bone metastasis ↑	^121^
circRBMS3	miR-654-3p	Bone metastasis ↑	^122^
lncRNA MALAT1	Tead3/Nfatc1	Bone metastasis ↓	^127^
lncRNA MIR193BHG	miR-489-3p/DNMT3A	Osteoclastogenesis ↑, Bone metastasis ↑	^35^
lncRNA SNHG3	miR-1273g-3p/BMP3	Bone metastasis ↑	^125^
lncRNA TRG-AS1	miR-877-5p/WISP2	Bone metastasis ↓	^126^
miR-10b	HOXD10/RhoC	Bone metastasis ↑	^141^
miR-124	IL-11/STAT3	Bone metastasis ↓	^134^
miR-135/miR-203	RUNX2	Bone metastasis ↓	^135^
miR-214-3p	Traf3	Bone resorption ↑, Bone metastasis ↑	^132^
miR-218-5p	DKK1/SFRP1	Bone metastasis ↑	^133,142^
miR-223-3p	MDK	Bone metastasis ↓	^140^
miR-30s	IL-11/TWIST1, DKK1, RUNX2, CDH11, CTGF, ITGA5/ITGB3	Osteomimicry ↓, Bone metastasis ↓	^136^
miR-34a-5p	c-Src	Bone metastasis ↓	^137^
miR-429	CrkL/MMP-9	Bone resorption ↓, Bone metastasis ↓	^138^
miR-506	NFATc1	Bone metastasis ↓	^139^

## Therapeutic landscape of breast cancer bone metastasis

### Current standards of care for breast cancer bone metastasis

Currently, inhibiting osteoclast function and reducing bone resorption to break the “vicious cycle” is the only way to treat breast cancer-related bone events.^143^ The NCCN Clinical Practice Guidelines in Oncology (Bone Cancer, Version 2.2025) focus on primary bone cancers and locally aggressive giant cell tumor of bone (GCTB), with clear recommendations for denosumab and bisphosphonates regarding GCTB management, SREs prevention, and supportive care. Denosumab is mainly indicated for GCTB and secondarily for metastatic bone disease complications, bisphosphonates for metastatic primary bone cancer skeletal support, and GCTB second-line treatment. For primary bone cancers, denosumab is prioritized for GCTB, and bisphosphonates for metastatic SRE prevention. Denosumab needs no dose adjustment (Category 1 Preferred in eGFR <30 mL/min); bisphosphonates are avoided/reduced in severe renal dysfunction. Both carry ONJ (~1%–3%) and hypocalcemia risks (denosumab > bisphosphonates), denosumab has lower renal toxicity, and bisphosphonates have higher acute-phase reactions. For GCTB, denosumab should be tapered or followed by bisphosphonates to avoid rebound bone resorption. Bisphosphonates can be discontinued after 2–3 years of stable disease.^144^

Denosumab and bisphosphonates are recognized as standard bone-modifying drugs for malignant tumor bone metastasis, strongly recommended (Grade I/A) by international guidelines like the 5th ESO-ESMO International Consensus Guidelines for Advanced Breast Cancer, which emphasizes their routine combination with systemic therapy to prevent SREs and notes denosumab’s priority for bisphosphonate-intolerant or renal dysfunction patients due to its kidney-sparing property.^145^ As the first and only fully human monoclonal antibody targeting RANKL, denosumab exerts superior efficacy and safety by blocking osteoclast signaling, serving as a key alternative for patients unresponsive or intolerant to bisphosphonates.^146^ A phase III subgroup analysis confirmed its biosimilar QL1206 matches the original drug in efficacy, safety, and bone metabolism markers,^146^ while a meta-analysis of 11 randomized controlled trials further validated denosumab’s lower acute-phase reaction risk, especially for renal-impaired patients.^147^

Bisphosphonates exert anti-resorptive effects by inhibiting farnesyl pyrophosphate synthase, thereby inducing osteoclast apoptosis and reducing bone resorption.^148^ Denosumab, a fully humanized monoclonal antibody against RANKL, directly blocks the interaction between RANKL and its receptor RANK on osteoclast precursors, inhibiting osteoclast differentiation, maturation, and survival.^149^ Compared with bisphosphonates, denosumab shows superior efficacy in delaying first SREs and reducing bone turnover markers in metastatic bone disease, and is recommended as a first-line option for patients intolerant to bisphosphonates.^150^ Above all, denosumab and bisphosphonates jointly constitute the standard bone-modifying therapeutic drugs in the field of malignant tumor bone metastasis.

In the early stages of breast cancer, bisphosphonates are used to prevent or mitigate the adverse effects of adjuvant endocrine therapy on the skeleton.^151^ By limiting bone resorption, they relieve the pain of patients with bone metastases, reduce fractures and the development of new bone lesions, and thus improve the quality of life of patients.^152,153^ However, several studies have indicated that bisphosphonates and denosumab fail to improve overall survival in breast cancer patients with bone metastases.^9^ An observation partly attributed to their association with medication-related osteonecrosis of the jaw (MRONJ). MRONJ refers to the occurrence of jaw necrosis during the extensive use of anti-resorptive drugs such as bisphosphonates and denosumab, or targeted drugs such as angiogenesis inhibitors for the treatment of osteoporosis and prevention of tumor bone metastases.^154^ Although these drugs inhibit osteoclast activity, long-term use may disrupt the microenvironment of the jaw, leading to jaw necrosis, which may be related to the inhibition of normal osteoclastogenesis and signaling pathways.^155^ While MRONJ does not directly cause patient death, it exerts indirect yet impactful effects on survival prognosis by disrupting treatment continuity, exacerbating comorbidities, and compromising nutritional status and quality of life, key factors that collectively influence the overall clinical outcomes of this patient population.^154,156,157^

Radiotherapy is still a viable treatment option for some breast cancer patients with bone metastases. For ER-positive and HER2-negative advanced breast cancer patients with bone metastases who received CDK4/6 inhibitor treatment, the median overall survival of those who received bone radiotherapy was 49.1 months, compared with 40.5 months for those who did not.^158^ Radium-223, as a bone-targeted radiopharmaceutical,^159^ cause irreversible double-strand breaks in the DNA of tumor cells through the generation of high-energy, short-range α particles, thereby inducing tumor cell death and selectively targeting the areas of bone metastases.^160^ In HR-positive breast cancer patients with bone metastases, the combination of radium-223 and endocrine therapy has shown significant efficacy.^161^

### Next-generation therapies targeting breast cancer bone metastasis

Due to the significant drug resistance in breast cancer current therapy, a thorough investigation into the molecular mechanisms underlying the synergistic effect may provide a direction for novel therapeutic targets in breast cancer treatment.^162–164^ In the initial stage of classical osteoclastogenesis, RANKL binds to its receptor RANK, activating the NF-κB and c-Fos signaling pathways, which in turn promotes the early expression and nuclear translocation of NFATc1.^165^ In the middle and late stages of osteoclastogenesis, NFATc1 stimulates the expression of a series of key genes for osteoclast development, including *NFATc1*, *c-Src*, *Integrin β3*, *Mmp9*, *Trap*, and *Ctsk*, ultimately leading to the maturation of osteoclasts.^166^ The NFATc1 inhibitor erianin effectively inhibits the formation of osteoclasts and reduces the osteolytic damage caused by breast cancer.^167^ Recent preclinical studies demonstrate that it directly binds to NFATc1 to suppress the expression of osteoclast-specific genes (e.g., *TRAP*, *CTSK*), thereby inhibiting breast cancer cell-induced osteoclast activation and bone destruction.^167^ In vitro experiments show erianin reduces osteoclast formation rate by 58.2% at 100 nmol/L, and in vivo mouse models confirm it attenuates bone metastasis lesions without obvious systemic toxicity.^167^ However, no clinical trials have been initiated to verify its safety and efficacy in humans. In addition, human monoclonal antibody targeting Jagged1 not only blocks tumor-induced osteolysis, but also enhances the sensitivity of tumor cells to chemotherapy.^168^ Cathepsin K inhibitor L-235 effectively inhibits tumor-induced osteolytic damage and significantly reduces the skeletal tumor burden.^169^ Saracatinib (AZD0530), as a Src kinase inhibitor, effectively reduces bone resorption in patients with advanced malignant tumors by inhibiting the activity of osteoclasts, thereby intervening in the process of bone metastasis.^170^ The kinase regulation mechanism can help identify key nodes that can be targeted by drugs, and provide more options for the treatment of metastatic cancer.^171^

Phenotypic plasticity is considered a key driving force for normal development, tumorigenesis, and tumor progression.^172,173^ The N6-methyladenosine (m6A) reader YTHDF1 promotes osteolytic bone metastases of breast cancer by inducing EZH2 and CDH11 translation.^174^ The bone microenvironment induces reversible phenotypic conversion of ER-positive breast cancer cells through EZH2-mediated epigenetic reprogramming, leading to endocrine resistance. This study provides a treatment strategy of “environment-epigenetic-metabolism”.^175^ In addition, a novel nano-drug is based on phosphate ion-responsive and calcium peroxide-containing nanoparticles, which carry the bone-targeting agents and photosensitizers. These nanoparticles effectively accumulate in bone metastatic lesions, suppressing tumor growth and repairing osteolytic bone damage, thus offering a novel therapeutic strategy.^176^ The pH-sensitive dual ligand-targeted polymeric micelles P123-ALN/DP-8@DOX also successfully treat BCBM.^177^ Furthermore, targeting RNA helicase DDX3 may serve either as monotherapy or in combination with standard-of-care therapeutics for the treatment of BCBM.^178^ The interaction between the intratumoral microbiota and cancer immunotherapy also provides a novel therapeutic strategy for BCBM.^179^

Additionally, other early-phase strategies include targeted therapies against key signaling pathways in the bone metastasis niche (e.g., Wnt signaling inhibitors), immunotherapies, and novel antibody-drug conjugates (ADCs) with bone-tropism, which are being explored to enhance tumor-specific killing while modulating the bone microenvironment. RSPO2 and RANKL, through the common receptor LGR4, regulate the expression of Wnt inhibitor DKK1 via Gαq and β-catenin signaling. This, in turn, promotes the formation of PMNs for osteoclasts and enhances BCBM. LGR4 and DKK1 are promising novel targets for intervention in bone metastasis.^180^ ICB combined with denosumab can significantly reverse this resistance and prolong the progression-free survival of breast cancer patients with bone metastasis.^107^ Bone-targeted modified antibody therapy achieves specific enrichment of BCBM by targeting specific targets in the bone microenvironment, significantly enhancing the anti-tumor activity of antibody therapy while reducing systemic toxicity. This provides a novel strategy for the precise targeted treatment of BCBM.^181^ The combination of the flavonoid compound ononin and the JNK inhibitor synergistically enhances the inhibitory effect on BCBM, providing novel potential drugs and strategies for the treatment of BCBM.^182^ These experimental approaches are still in the preliminary stages of clinical development, with ongoing studies focusing on optimizing dosing, evaluating safety profiles, and validating efficacy in BCBM populations.

## Conclusions and future prospects

**Fig. 6:**
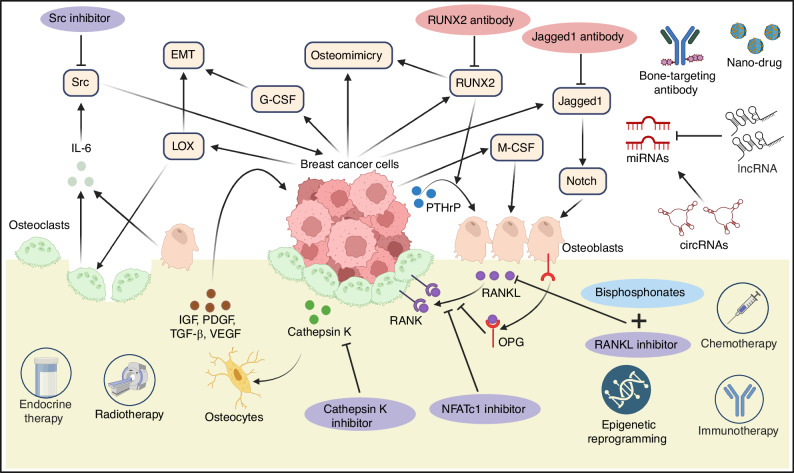
The vicious cycle of bone metastasis in breast cancer and its treatment. Breast cancer cells colonize in the bones, release various osteomimetic factors, which promote the activation of osteoblasts. Osteoblasts release RANKL, which binds to RANK on osteoclasts, activates the osteoclasts. Osteoclasts dissolve bone minerals and degrade the collagen fibers of the bone matrix by secreting cathepsin K and MMPs, leading to bone resorption. During this process, a large number of growth factors are released. These growth factors not only directly promote the proliferation of tumor cells, but also stimulate tumor cells to further secrete osteomimetic factors, forming a vicious cycle, which continuously exacerbates bone destruction and tumor progression. The corresponding targeted therapy can effectively prevent the occurrence of bone metastasis in breast cancer. In addition, radiation therapy, chemotherapy, endocrine therapy, immunotherapy, nano-drugs and epigenetic programming therapies all play significant roles. Among them, circRNAs and miRNAs play an important regulatory role in the process of bone metastasis

In conclusion, bone metastasis is a highly prevalent clinical event in patients with breast cancer. The crosstalk among tumor cells, host cells, and the bone microenvironment plays a critical role in the progression of breast cancer-induced osteolytic lesions. Upon entering the bone microenvironment, metastatic breast cancer cells secrete a variety of osteomimetic factors, which stimulate osteoblasts and enhance the expression of RANKL. The process of bone resorption mediated by osteoclasts highly depends on the interaction between RANK on their surface and its ligand RANKL. At the same time, osteoblasts secrete OPG, which antagonize the bone resorption mediated by osteoclasts, thus maintaining the balance of bone metabolism. However, once this balance is disrupted, over-activated osteoclasts will degrade the bone matrix by producing strong acids and proteases, such as cathepsin K and MMPs. This process will lead to the release of growth factors stored in the bone matrix, which in turn promotes the growth of tumor cells. Moreover, tumor growth will further increase the level of tumor-derived PTHrP, forming a vicious cycle. This vicious cycle accelerates the interaction between tumor cells and the matrix in the bone microenvironment, creating extremely favorable conditions for the invasive behavior of malignant tumor cells that have entered the bone microenvironment. In addition, LOX secreted by tumor cells act in synergy with RANKL to mediate the differentiation of osteoclasts, further accelerating the bone resorption by osteoclasts and promoting the formation of metastatic osteolytic lesions. Notably, the ncRNAs also play essential regulatory roles in this vicious cycle. These findings provide a valuable theoretical framework and point to promising directions for future research into the mechanisms of BCBM and the identification of novel therapeutic targets (Fig. 6).
